# Comorbidities and use of health services in people with diabetes mellitus according to risk levels by adjusted morbidity groups

**DOI:** 10.1186/s12902-024-01634-0

**Published:** 2024-07-16

**Authors:** Jaime Barrio-Cortes, María Pilar Mateos-Carchenilla, María Martínez-Cuevas, María Teresa Beca-Martínez, Elvira Herrera-Sancho, María Carmen López-Rodríguez, María Ángeles Jaime-Sisó, Montserrat Ruiz-López

**Affiliations:** 1 Foundation for Biosanitary Research and Innovation in Primary Care, Madrid, Spain; 2 Primary Care Investigation Unit, Gerencia Asistencial de Atención Primaria, Madrid, Spain; 3 Faculty of Health, Universidad Camilo José Cela, Madrid, Spain; 4 V Centenario Healthcare Centre, Gerencia Asistencial de Atención Primaria, San Sebastián de los Reyes, Madrid, Spain; 5 Fuencarral Healthcare Centre, Madrid Health Service, Madrid, Spain; 6 Ciudad Jardín Healthcare Centre, Gerencia Asistencial de Atención Primaria, Madrid, Spain; 7 Nursing School, Fundación Jiménez Diaz Hospital, Universidad Autónoma de Madrid, Madrid, Spain

**Keywords:** Diabetes mellitus, Comorbidities, Health services utilisation, Primary care, Hospital care, Adjusted morbidity groups

## Abstract

**Background:**

People with diabetes mellitus frequently have other comorbidities and involve greater use of primary and hospital care services. The aim of this study was to describe the comorbidities and use of primary and hospital care services of people with diabetes according to their risk level by adjusted morbidity groups (AMG) and to analyse the factors associated with the utilisation of these services.

**Methods:**

Cross-sectional study. People with diabetes were identified within the population of patients with chronic conditions of an urban health care centre by the AMG stratification tool integrated into the primary health care electronic clinical record of the Community of Madrid. Sociodemographic, functional, clinical characteristics and annual health care services utilisation variables were collected. Univariate, bivariate and Poisson regression analyses were performed.

**Results:**

A total of 1,063 people with diabetes were identified, representing 10.8% of patients with chronic conditions within the health centre. A total of 51.4% were female, the mean age was 70 years, 94.4% had multimorbidity. According to their risk level, 17.8% were high-risk, 40.6% were medium-risk and 41.6% were low-risk. The most prevalent comorbidities were hypertension (70%), dyslipidaemia (67%) and obesity (32.4%). Almost 50% were polymedicated. Regarding health services utilisation, 94% were users of primary care, and 59.3% were users of hospital care. Among the main factors associated with the utilisation of both primary and hospital care services were AMG risk level and complexity index. In primary care, utilisation was also associated with the need for primary caregivers, palliative care and comorbidities such as chronic heart failure and polymedication, while in hospital care, utilisation was also associated with comorbidities such as cancer, chronic obstructive pulmonary disease or depression.

**Conclusions:**

People with diabetes were older, with important needs for care, many associated comorbidities and polypharmacy that increased in parallel with the patient’s risk level and complexity. The utilisation of primary and hospital care services was very high, being more frequent in primary care. Health services utilization were principally associated with functional factors related to the need of care and with clinical factors such as AMG medium and high-risk level, more complexity index, some serious comorbidities and polymedication.

**Supplementary Information:**

The online version contains supplementary material available at 10.1186/s12902-024-01634-0.

## Background

In the Western world, population ageing, economic development, unhealthy diet and sedentary lifestyles have contributed to the increase in chronic diseases such as diabetes mellitus [1–4]. These types of diseases do not occur in isolation and are often associated, triggering numerous complications, as well as the needs of follow-up and care [5–7]. It is estimated that there are more than 500 million diabetic people worldwide, and various studies foresee a twofold increase in its prevalence in 2045 [1]. In Spain, data obtained in recent studies show prevalences between 11% and 14% [8, 9], with people with diabetes becoming one of the ten main causes of complications and death [10, 11].

Among the comorbidities most frequently associated in people with diabetes are cardiovascular diseases such as hypertension, coronary heart disease, congestive heart failure, cardiac arrhythmias, cerebrovascular disease or valvular heart disease. Other common associated comorbidities are obesity, chronic kidney failure, anaemia, cancer, liver disease, chronic obstructive pulmonary disease, cataracts and hypothyroidism. There are also psychiatric disorders, such as depression, alcohol abuse and other substance abuse [5, 12, 13]. The approach to these comorbidities must integrate multimorbidity, which is the simultaneous presence of 2 or more chronic diseases in a patient that require a comprehensive and multidisciplinary approach [14–16]. In addition to this frequent association with other comorbidities, diabetes leads to greater use of primary and hospital care services [17–20].

Morbidity groupers such as Clinical Risk Group (CRG) or Adjusted Clinical Groups (ACG) are used to stratify the population and optimise clinical-care management, favouring a better distribution of resources and a more efficient and personalised use of health care services [21]. In recent years, Adjusted Morbidity Groups (AMG) have been integrated in the electronic clinical records of several autonomous communities in Spain within the regional care strategies for patients with chronic conditions. This was aimed to stratify chronic patients according to their risk levels and to allow health professionals to choose different levels of intervention and subsequent specific care plans for the patients based on their clinical situation [22]. AMG are a multimorbidity measurement system that allows stratification of patients with chronic diseases, taking in consideration the patient’s comorbidity burden, into different risk levels following the Kaiser Permanente pyramid model (chronic patients with high risk, medium risk or low risk) [23]. The low-risk patients (in the base of the pyramid) have a lower health care services and needs; prevention and health promotion measures are focused on empowering patients and self-management. The medium-risk patients (in the middle of the pyramid) have a greater need for care; they are managed according to their diseases, alternating self-care with use of health services, mainly primary care services, but also hospital care when is needed. The high-risk patients (at the top of the pyramid) have the greatest consumption of resources and needs of care are and measures are directed towards case management, relying most of their care in primary care and hospital level [24]. Thus, the use of AMG helps health personnel to better identify patients with greater comorbidities, risk of complications, worse quality of life and greater health care needs to develop individualized actions at each level of care according to the characteristics of the patient [25].

The aim of this study was to describe the comorbidities and use of primary care and hospital care services of people with diabetes according to risk level by AMG and to analyse the factors associated with the utilisation of these services.

## Methods

### Design

Cross-sectional descriptive study.

### Setting and study population

The study population was selected from an urban health centre with a total of 9,866 patients with chronic conditions. This Health Centre was in the north district of the city of Madrid. These patients have a tertiary university hospital for referrals. The district had a population of 143,424 people, with 55% women, an average age of 45 years, 8.9% with foreign nationality and the lowest degree of deprivation in Madrid based on the Medea index [26].

### Study subjects

**Fig. 1:**
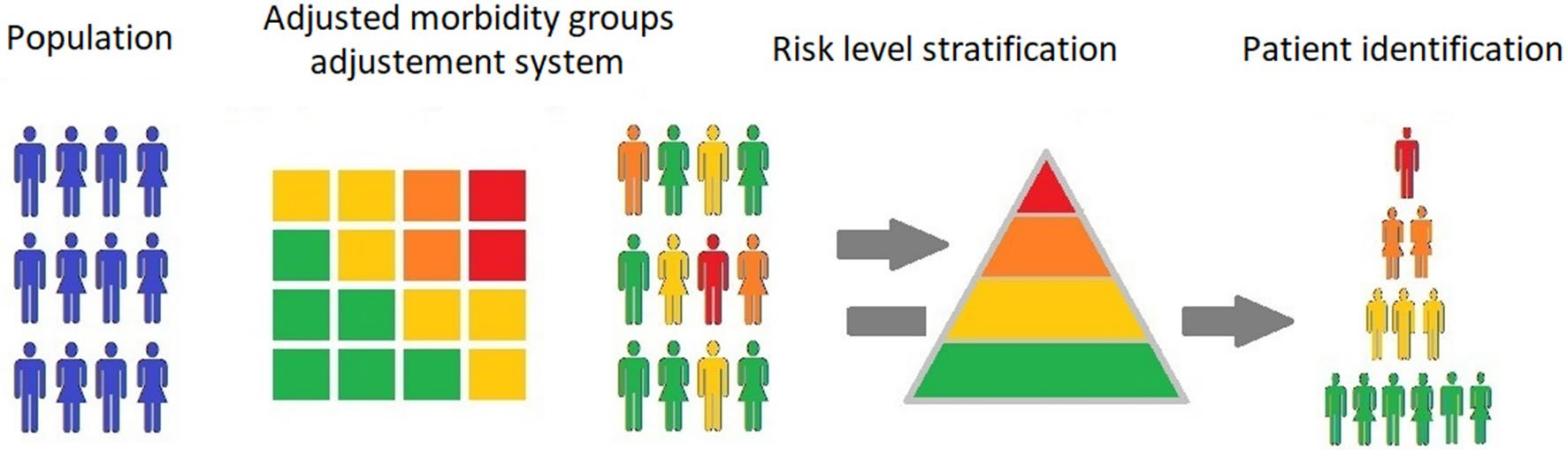
Adjusted morbidity groups measurement system and risk levels

The subjects studied were people with diabetes identified among the chronic patients assigned to the health centre through the AMG stratification tool integrated into the electronic medical record (EMR) of the Madrid Primary Care System. The AMG stratifies the population into mutually exclusive groups based on the diagnostic codes recorded in the EMR for each patient by the health professionals responsible for their care and different variables, such as risk of mortality, admissions, visits to primary care, and prescriptions, assigning the patient a numerical value (complexity index). This index, allows the population to be stratified into three risk levels (high, medium, and low risk) as well as the subset without relevant chronic pathology by assigning four cut-off points obtained from the 40th, 70th, 85th, and 95th percentiles of the entire population (Fig. 1) [27, 28].

The diseases that are considered as chronic by the AMG morbidity grouper are based on the Strategy of care for patients with chronic diseases of the Community of Madrid [25] and are shown in *Additional file 1.*

### Variables

The dependent variables were 1) the utilisation of primary care services per year: (a) total number of contacts, (b) type of contact (administrative, laboratory or health), (c) form of contact (face to face, telephone, home visit] and d) specialisation of the contacted professional (nurse, family doctor, physiotherapist, midwife, dentist and social worker) and 2) the utilisation of hospital care services per year (emergency room visits, outpatient visits, hospital admissions and day-care hospital visits). The independent variables were (1) sociodemographic variables (age, sex and country of origin), (2) functional variables (immobilised at home, institutionalized in a nursing home/retirement home due to severe immobility/functional impairment, need for a primary caregiver due to their situation of dependence, home support and palliative care) [30], and (3) clinical variables (risk level according to AMG and complexity index [a numerical value of patient complexity assigned by AMG, which is an index measured as a function of morbidity and health service utilisation] [27, 28], number and type of chronic diseases, multimorbidity, and polymedication [patients with a medication regimen that implies having been prescribed five or more medications for their chronic conditions as a reference treatment]).

### Sources of information

The information was obtained from the data recorded in the EMR of the Madrid Primary Care System. Sociodemographic, functional, and clinical variables were recorded as of June 30, 2015, and the annual study period of health services utilisation was between June 30, 2015, and June 30, 2016.

### Statistical analysis

A descriptive analysis of the sociodemographic, functional, clinical and service utilisation variables in primary care and hospital was performed. Qualitative variables were described using frequencies and percentages, and quantitative variables were described using the mean and standard deviation. The distribution of continuous variables was analysed with the Kolmogorov‒Smirnov test. The association between the different variables was assessed by parametric or nonparametric tests based on the distribution of the variables. A posteriori contrast was performed. Multiple tests were adjusted by the Bonferroni method. To analyse the factors associated with the use of primary care and hospital care services, two Poisson regression models were constructed, one whose dependent variable was the number of annual total contacts with primary care and the other whose dependent variable was the number of annual total contacts with hospital care, while the independent variables in both were those that were significantly associated in the bivariate analysis and had clinical relevance. The results were considered as statistically significant if the p values were ≤ 0.05. Statistical analysis of the data was performed with the IBM SPSS Statistics Version 25 program.

## Results

A total of 1,063 people with diabetes were identified, which represented 10.8% of the total chronic patients of the health centre. Among these people with diabetes, 51.4% were women, the average age was 70 years, and 83.5% were of Spanish nationality. A total of 8.9% were immobilised at home, 7% needed a primary caregiver due to their dependency, 3% were institutionalized, 2.6% required home support, and 1.7% were receiving palliative care. Their differences in terms of sociodemographic and clinical characteristics compared to people without diabetes can be seen in Table 1.

**Table 1:** Sociodemographic and functional characteristics of the chronic patients in the study

Chronic patients *n* (%)	Total 9,866 (100)	Diabetic 1,063 (10.8)	95% CI	Nondiabetic 8,803 (89.2)	95% CI	*p*- value
Female	6,056 (61.4)	546 (51.4)	48.3–54.3	5,510 (62.6)	61.5–63.6	< 0.01
Age*	55.7 (20.8)	70.0 (14.8)	69.1–70.9	54.0 (20.7)	53.6–54.4	< 0.01
≤ 75 years old > 75 years old	7,896 (80) 1,970 (20)	624 (58.7) 439 (41.3)	55.7–61.7 38.3–44.2	7,272 (82.6) 1,531 (17.4)	81.8–83.4 16.6–18.2	< 0.01
Origin Spain Europe Rest of the World	8,078 (81.9) 367 (3.7) 1,421 (14.4)	888 (83.5) 34 (3.2) 141 (13.3)	81.3–85.8 2.1–4.3 13.8–15.8	7,190 (81.7) 333 (3.8) 1,280 (14.5)	80.8–82.5 3.4–4.2 13.8–15.3	0.3
Immobilised	300 (3.0)	95 (8.9)	7.2–10.6	205 (2.3)	2.0–2.6	< 0.01
Primary caregiver	229 (2.3)	74 (7.0)	5.4–8.4	155 (1.8)	1.5–2.0	< 0.01
Institutionalised in a nursing/ retirement home	161 (1.6)	32 (3.0)	2.0–4.0	129 (1.5)	1.2–1.7	< 0.01
Home support	80 (0.8)	28 (2.6)	1.7–3.6	52 (0.6)	0.4–0.7	< 0.01
Palliative care	44 (0.4)	18 (1.7)	0.9–2.4	26 (0.3)	0.1–0.4	< 0.01

* Mean (Standard deviation)

Regarding the clinical characteristics among people with diabetes, 17.8% had high risk, 40.6% had medium risk and 41% had low risk according to AMG, and 94.4% had multimorbidity. Among cardiovascular comorbidities, the most prevalent were hypertension (69.9%), dyslipidaemia (66.9%), dysrhythmias (15.6%), coronary heart disease (12.2%) and chronic heart failure (7.8%). Among the noncardiovascular comorbidities, the most prevalent were obesity (32.4%), thyroid disorder (19.9%), arthrosis (17.1%), anxiety (15.9%), depression (15.1%) and anaemia (10.8%). A total of 43.6% were polymedicated. Differences in their clinical characteristics compared to people without diabetes are shown in Table 2.

**Table 2:** Clinical characteristics of the chronic patients in the study

Chronic patients *n* (%)	Total 9,866 (100)	Diabetic 1,063 (10.8)	95% CI	Nondiabetic 8,803 (89.2)	95% CI	*p*- value
Risk level by AMG High Medium Low	444 (4.5) 1,784 (18.1) 7,638 (77.4)	189 (17.8) 432 (40.6) 442 (41.6)	15.5–20.0 38.6–42.6 37.7–43.6	255 (2.9) 1,352 (15.4) 7,196 (81.7)	2.5–3.2 14.6–16.0 81.0–82.5	< 0.01
Complexity index by AMG*	6.7 (7.0)	13.1 (10.1)	12.5–13.7	5.9 (6.1)	5.8–6.1	< 0.01
Multimorbidity	6,036 (61.2)	1,033 (94.4)	92.9–95.7	5,033 (57.2)	56.0–58.0	< 0.01
Chronic diseases*	2.5 (1.8)	4.7 (2.3)	4.6–4.9	2.3 (1.6)	2.2–2.4	< 0.01
Alcohol abuse	407 (4.1)	56 (5.3)	3.9–6.6	351 (4.0)	3.5–4.4	0.047
Anaemia	908 (9.2)	111 (10.4)	8.6–12.3	797 (9.1)	8.4–9.6	0.14
Aortic Aneurism	47.0 (0.5)	10 (0.9)	0.3–1.5	37.0 (0.4)	0.2–0.5	0.02
Anxiety	2,345 (23.8)	169 (15.9)	13.7–18.1	2,176 (24.7)	23.8–25.6	< 0.01
Arthritis	235 (2.4)	30 (2.8)	1.8–3.8	205 (2.3)	2–2.6	0.32
Arthrosis	1,055 (10.7)	182 (17.1)	14.8–19.4	873 (9.9)	9.3–10.5	< 0.01
Asthma	1,044 (10.6)	56 (5.3)	4.0–7.0	988 (11.2)	10.5–11.8	< 0.01
Cancer	481 (4.9)	90 (8.5)	6.8–10.1	391 (4.4)	4.0–4.8	< 0.01
Cirrhosis	479 (4.9)	113 (10.6)	8.8–12.5	366 (4.2)	3.7–4.5	< 0.01
Coronary heart disease	370 (3.8)	130 (12.2)	10.2–14.2	240 (2.7)	2.3–3.1	< 0.01
Chronic heart failure	240 (2.4)	83 (7.8)	6.1–9.4	157 (1.8)	1.5–2.1	< 0.01
Chronic renal failure	142 (1.4)	62 (5.8)	4.4–7.2	80 (0.9)	0.7–1.1	< 0.01
COPD	389 (3.9)	87 (8.2)	6.5–9.8	302 (3.4)	3.0–3.8	< 0.01
Dementia	213 (2.2)	40 (3.8)	2.6–4.9	173 (2.0)	1.6–2.3	< 0.01
Depression	1,251 (12.7)	160 (15.1)	12.9–17.2	1,091 (12.4)	11.7–13.1	0.014
Dyslipidaemias	3,780 (38.3)	711 (66.9)	64.1–69.7	3,069 (34.9)	33.8–35.8	< 0.01
Dysrhythmias	696 (7.1)	166 (15.6)	13.4–17.8	530 (6.0)	5.5–6.5	< 0.01
Epilepsy	187 (1.9)	10 (0.9)	0.1-2	177 (2.0)	1.7–2.3	0.016
Femur fracture	13 (0.1)	2 (0.2)	0.04–0.4	11 (1.0)	0.5–1.5	0.59
Glaucoma	395 (4.0)	71 (6.7)	5.1–8.2	324 (3.7)	3.2–4.1	< 0.01
Hypertension	3,418 (34.6)	743 (69.9)	67.1–73.6	2,675 (30.4)	29.4–31.3	< 0.01
HIV	55 (0,6)	4 (0.4)	0.1–0.7	51 (0,6)	0.4–0.8	0.40
Inflammatory bowel disease	75 (0.8)	4 (0.4)	0.1–0.7	71 (0.8)	0–1.9	0.13
Obesity	1,626 (16.5)	344 (32.4)	29.5–35.2	1,282 (14.6)	13.8–15.3	< 0.01
Osteoporosis	1,113 (11.3)	128 (12.0)	10.0–14.0	985 (11.2)	10.5–11.8	< 0.01
Parkinson	85 (0.9)	16 (1.5)	0,8–2,2	69 (0.8)	0,6–1.0	0.016
Repeat urinary infections	497 (5)	66 (6.2)	4.7–7.7	431 (4.9)	4.4–5.5	0.06
Stroke	267 (2.7)	65 (6.1)	4.7–7.5	202 (2.3)	1.9–2.6	< 0.01
Thyroid disorder	1,646 (16.7)	212 (19.9)	17.5–22.3	1,434 (16.3)	15.5–17.1	< 0.01
Valvular heart disease	196 (2)	39 (3.7)	3.1–5.2	157 (1.8)	1.5–2.0	< 0.01
Polymedicated	1,598 (16.2)	463 (43.6)	40.6–46.5	1,135 (12.9)	12.2–13.4	< 0.01

* Mean (Standard deviation). AMG: Adjusted morbidity groups. COPD: Chronic obstructive pulmonary disease

The sociodemographic and functional characteristics of people with diabetes by risk level according to AMG and by sex and their differences are shown in Table 3.

**Table 3:** Sociodemographic and functional characteristics of people with diabetes by risk level according to AMG and by sex

People with diabetes *n* (%)	High risk 189 (17.8)	Medium risk 432 (40.6)	Low risk 442 (41.6)	*p*- value	Female 546 (51.4)	Male 517 (48.6)	*p*- value
Female	92 (48.7)	232 (53.7)	222 (50.02)	0.42	546 (100)	0 (0)	-
Age*	77.5 (10.2)	74.3 (11.8)	62.6 (15.9)	< 0.01	71.1 (15.7)	68.9 (13.8)	< 0.01
≤ 75 years old > 75 years old	67 (35.4) 122 (64.6)	206 (47.7) 226 (52.3)	351 (79.4) 91 (20.6)	< 0.01	287 (52.6) 259 (47.4)	337 (65.2) 180 (34.8)	< 0.01
Origin Spain Europe Rest of the World	162 (85.7) 3 (1.6) 24 (12.7)	372 (86.1) 15 (3.5) 45 (10.4)	354 (80.1) 16 (3.6) 72 (16.3)	0.07	449 (82.2) 18 (3.3) 79 (14.5)	439 (84.9) 16 (3.1) 62 (12.0)	0.23
Immobilised	47 (24.9)	38 (8.8)	10 (2.3)	< 0.01	66 (12.1)	29 (5.6)	< 0.01
Institutionalized in a nursing/ retirement home	15 (7.9)	14 (3.2)	3 (0.7)	< 0.01	17 (3.1)	15 (2.9)	0.84
Primary caregiver	40 (21.2)	29 (6.7)	5 (1.1)	< 0.01	53 (9.7)	21 (4.1)	< 0.01
Home support	12 (6.3)	11 (2.5)	5 (1.1)	< 0.01	19 (3.5)	9 (1.7)	0.08
Palliative care	14 (7.4)	1 (0.2)	3 (0.7)	< 0.01	7 (1.3)	11 (2.1)	0.29

* Mean (Standard deviation)

The clinical characteristics of people with diabetes by risk level according to AMG and by sex and their differences are shown in Table 4.

**Table 4:** Clinical characteristics of people with diabetes by risk level according to AMG and by sex

People with diabetes *n* (%)	High risk 189 (17.8)	Medium risk 432 (40.6)	Low risk 442 (41.6)	*p*- value	Female 546 (51.4)	Male 517 (48.6)	*p*- value
Complexity index by AMG*	30.5(11.3)	12.9 (2.6)	5.8 (2.1)	< 0.01	12.74 (9.3)	13.5 (10.9)	0.80
Multimorbidity	189 (100)	432 (100)	382 (86.4)	< 0.01	520 (95.2)	483 (93.4)	0.20
Chronic diseases*	7.7 (2.2)	5.2 (1.4)	3.1 (1.3)	< 0.01	5 (2.3)	4.4 (2.2)	< 0.01
Alcohol abuse	16 (8.5)	26 (6.0)	14 (3.2)	0.016	9 (1.6)	47 (9.1)	< 0.01
Anaemia	48 (25.4)	42 (9.7)	21 (4.8)	< 0.01	68 (12.5)	43 (8.3)	0.027
Anxiety	34 (18.0)	73 (16.9)	62 (14.0)	0.35	114 (20.9)	55 (10.6)	< 0.01
Aortic aneurysm	7 (3.7)	3 (0.7)	0 (0)	< 0.01	1 (0.2)	9 (1.7)	< 0.01
Arthritis	8 (4.2)	20 (4.6)	2 (0.5)	< 0.01	19 (3.5)	11 (2.1)	0.18
Arthrosis	45 (23.8)	106 (24.5)	31 (7.0)	< 0.01	130 (23.8)	52 (10.1)	< 0.01
Asthma	12 (6.3)	29 (6.7)	15 (3.4)	0.06	48 (8.8)	8 (1.5)	< 0.01
Cancer	52 (27.5)	34 (7.9)	4 (0.9)	< 0.01	31 (5.7)	59 (11.4)	< 0.01
Cirrhosis	27 (14.3)	66 (15.3)	20 (4.5)	< 0.01	64 (11.7)	49 (9.5)	0.23
Chronic heart failure	52 (27.5)	28 (6.5)	3 (0.7)	< 0.01	46 (8.4)	37 (7.2)	0.44
Chronic renal failure	51 (27.0)	11 (2.5)	0 (0)	< 0.01	27 (4.9)	35 (6.8)	0.20
Coronary heart disease	65 (34.4)	53 (12.3)	12 (2.7)	< 0.01	41 (7.5)	89 (17.2)	< 0.01
COPD	49 (25.9)	30 (6.9)	8 (1.8)	< 0.01	24 (4.4)	63 (12.2)	< 0.01
Dementia	21 (11.1)	17 (3.9)	2 (0.5)	< 0.01	28 (5.1)	12 (2.3)	0.016
Depression	43 (22.8)	85 (19.7)	32 (7.2)	< 0.01	112 (20.5)	48 (9.3)	< 0.01
Dyslipidaemias	155 (82.0)	329 (76.2)	227 (51.4)	< 0.01	363 (66.5)	348 (67.3)	0.78
Dysrhythmias	85 (45.0)	65 (15.0)	16 (3.6)	< 0.01	84 (15.4)	82 (15.9)	0.83
Epilepsy	5 (2.6)	4 (0.9)	1 (0.2)	0.01	3 (0.5)	7 (1.4)	0.20
Femur fracture	2 (1.1)	0 (0)	0 (0)	0.01	2 (0.4)	0 (0)	0.17
Glaucoma	26 (13.8)	36 (8.3)	9 (2.0)	< 0.01	45 (8.2)	26 (5.0)	0.04
Hypertension	171 (90.5)	350 (81.0)	222 (50.2)	< 0.01	376 (68.9)	367 (71.0)	0.45
HIV	3 (1.6)	1 (0.2)	0 (0)	0.01	1 (0.2)	3 (0.6)	0.29
Inflammatory bowel disease	0 (0)	3 (0.7)	1 (0.2)	0.34	2 (0.4)	2 (0.4)	0.96
Obesity	79 (41.8)	160 (37.0)	105 (23.8)	< 0.01	192 (35.2)	152 (29.4)	0.04
Osteoporosis	38 (20.1)	61 (14.1)	29 (6.6)	< 0.01	121 (22.2)	7 (1.4)	< 0.01
Parkinson	9 (4.8)	6 (1.4)	1 (0.2)	< 0.01	7 (1.3)	9 (1.7)	0.54
Repeated urinary infections	29 (15.3)	28 (6.5)	9 (2)	< 0.01	44 (8.1)	22 (4.3)	0.01
Stroke	33 (17.5)	28 (6.5)	4 (0.9)	< 0.01	30 (5.5)	35 (6.8)	0.39
Thyroid disorder	54 (28.6)	100 (23.1)	58 (13.1)	< 0.01	149 (27.3)	63 (12.2)	< 0.01
Valvular heart disease	32 (16.9)	6 (1.4)	1 (0.2)	< 0.01	20 (3.7)	19 (3.7)	0.99
Polymedicated	170 (89.9)	226 (52.3)	67 (15.2)	< 0.01	267 (48.9)	196 (37.9)	< 0.01

* Mean (Standard deviation). AMG: Adjusted morbidity groups. COPD: Chronic obstructive pulmonary disease

Regarding the use of health services, 94.6% of these people with diabetes used primary care services, and 59.3% used hospital care services, with an average of 22.4 annual contacts in primary care and 6.2 annual contacts in hospital care.

In relation to the type of contact with primary care, there was an average of 19.2 visits to health professionals compared to 1.8 administrative contacts and 1.4 contacts with the laboratory. Of these contacts, an average of 19.7 were in person, while there was a mean of 1.0 telephone contacts and 1.7 home visits.

In relation to the professionals contacted, the doctor was the most contacted with an average of 10.5 visits/year, followed by the nurse with 8.0 visits/year, physiotherapist with 0.3 visits/year and social worker with 0.2 visits/year.

Regarding hospital care, the most commonly used service was outpatient consultation, with an average of 4.6 visits/year, followed by 0.9 emergency room visits/year, 0.3 hospital admissions/year and 0.5 hospital day-care visits/year.

The use of primary and hospital care services of the total number of people with diabetes and segmented by AMG risk level, sex and age ≤/>75 years is shown in Table 5.

**Table 5:** Annual use of services of people with diabetes and according to risk level, sex and age groups

Primary care contacts Mean (Standard deviation)	People with diabetes 1,004 (94.4%)	95% CI	High risk 187 (98.9%)	Medium risk 425 (98.4%)	Low risk 392 (88.7%)	*p*- value	Female 515 (94.3%)	Male 489 (94.6%)	*p*- value	≤ 75 years old 577 (92.5%)	> 75 years old 427 (97.3%)	*p*- value
***Total annual contacts***	22.4 (20.4)	21.2–23.7	35.1 (31.0)	23.9 (18.1)	14.8 (12.9)	< 0.01	23.4 (20.2)	21.4 (17.0)	< 0.01	18.8 (16.0)	27.3 (23.4)	< 0.01
***Contact type***												
Health-related	19.2 (18.3)	18.1–20.4	30.1 (27.3)	20.5 (16.1)	12.7 (11.2)	< 0.01	20.2 (18.0)	18.3 (18.7)	< 0.01	16.1 (14.9)	23.5 (21.5)	< 0.01
Administrative	1.8 (4.7)	1.5–2.1	3.0 (6.3)	2.0 (5.0)	0.9 (3.2)	< 0.01	1.6 (4.4)	1.9 (5.1)	0.6	1.5 (4.2)	2.2 (5.3)	0.07
Laboratory	1.4 (1.7)	1.3–1.5	1.9 (2.2)	1.5 (1.6)	1.1 (1.4)	< 0.01	1.6 (1.9)	1.2 (1.5)	< 0.01	1.2 (1.4)	1.7 (2.0)	< 0.01
***Form of contact***												
Face-to-face	19.7 (16.0)	18.8–20.7	27.7 (19.9)	21.5 (15.3)	14.1 (12.4)	< 0.01	19.9 (15.1)	19.6 (17.0)	0.5	17.9 (15.9)	22.3 (16)	< 0.01
Telephone	1.0 (4.9)	0.7–1.3	2.6 (10.5)	0.9 (2.4)	0.3 (1.4)	< 0.01	1.3 (6.5)	0.7 (2.4)	0.04	0.5 (2.1)	1.7 (7.1)	< 0.01
Home visit	1.7 (6.2)	1.3–2.1	4.7 (9.9)	1.5 (6.3)	0.4 (1.8)	< 0.01	2.2 (6.8)	1.1 (5.4)	< 0.01	0.4 (2.8)	3.4 (8.7)	< 0.01
***Professional contacted***												
Family physician	10.5 (9.2)	10.0–11.1	16.7 (13.9)	11.2 (7.8)	6.9 (5.5)	< 0.01	11.4 (10.6)	9.7 (7.4)	< 0.01	8.9 (7.7)	12.8 (10.6)	< 0.01
Nurse	8.0 (11.6)	7.3–8.8	13.1 (18.4)	8.5 (10)	5.2 (7.3)	< 0.01	8 (9.0)	8.1 (13.8)	0.3	6.6 (9.2)	10.0 (14)	< 0.01
Physiotherapist	0.3 (2.1)	0.2–0.4	0.0 (0)	0.5 (2.7)	0.3 (1.8)	0.015	0.3 (2.1)	0.3 (2.1)	0.9	0.3 (1.9)	0.3 (2.3)	0.5
Midwife	0.07 (0.6)	0.04–0.1	0.0 (0.2)	0.0 (0.2)	0.1 (0.8)	< 0.01	0.1 (0.8)	0.0 (0)	< 0.01	0.1 (0.7)	0.0 (0.1)	< 0.01
Dentist	0.04 (0.3)	0.03–0.05	0.0 (0)	0.0 (0.3)	0.0 (0.3)	0.09	0.0 (0.2)	0.0 (0.5)	0.3	0.1 (0.4)	0.0 (0.1)	< 0.01
Social worker	0.2 (1.0)	0.1–0.3	0.3 (2.0)	0.2 (0.8)	0.1 (0.4)	< 0.01	0.2 (1.3)	0.1 (0.5)	0.01	0.1 (0.5)	0.3 (1.5)	< 0.01
**Hospital care contacts** Mean (Standard deviation)	**People with diabetes** 630 (59.3%)	**95% CI**	**High risk** 156 (82.5%)	**Medium risk** 296 (68.5%)	**Low risk** 178 (40.3%)	**P**	**Female** 308 (56.4%)	**Male** 322 (62.3%)	**p**	**≤ 75 years old** 577 (92.5%)	**> 75 years old** 427 (97.3%)	**p**
***Total annual contacts***	6.2 (7.8)	5.6–6.8	9.7 (12.4)	5.8 (5.5)	3.7 (3.4)	< 0.01	5.8 (6.0)	6.6 (9.2)	0.08	6.3 (9.0)	6.1 (6.0)	< 0.01
Outpatient consultation	4.6 (4.9)	4.2–4.9	6.3 (6.2)	4.7 (4.7)	2.8 (2.6)	< 0.01	4.2 (4.2)	4.9 (5.4)	0.05	4.7 (5.3)	4.4 (4.2)	0.03
Emergency	0.9 (1.4)	0.8–1.0	1.4 (1.8)	0.8 (1.2)	0.6 (1.0)	< 0.01	0.9 (1.4)	0.9 (1.4)	0.7	0.8 (1.3)	1.1 (1.5)	< 0.01
Hospitalisations	0.3 (0.7)	0.2–0.3	0.6 (1.0)	0.2 (0.5)	0.1 (0.4)	< 0.01	0.3 (0.7)	0.3 (0.7)	1	0.2 (0.7)	0.3 (0.7)	< 0.01
Day-care hospital	0.5 (3.4)	0.2–0.7	1.5 (6.5)	0.1 (0.9)	0.1 (0.8)	< 0.01	0.3 (2.3)	0.6 (4.2)	0.5	0.6 (4.3)	0.2 (1.7)	0.5

The multivariate analysis shown that factors that were positively associated with the use of primary care services were need for palliative care which increased 1.26 times the utilisation (Est.=0.23; 97.5% CI=[0.15, 0.31]) and need for primary caregiver which increased 1.39 times the utilisation (Est.=0.33; 97.5% CI=[0.29, 0.37]). Also, each point in complexity index increased 1.02 times the utilization of primary care services (Est.=0.02; 97.5% CI=[0.02, 0.02]), as well as medium and high risk level which increased 1.58 and 0.20 respectively the utilisation (Est.=0.23 and 0.06; 97.5% CI=[0.19 and 0.00; 0.26 and 0.13]). Others factors which increased 1.22 and 1.28 times this primary care utilisation were chronic heart failure (Est.=0.20; 97.5% CI=[0.15; 0.24]), and polymedication (Est.=0.25; 97.5% CI=[0.22; 0.29]). In contrast, the factors positively associated with the use of hospital care services were complexity index increasing 1.02 times with each point (Est.=0.02; 97.5% CI=[0.02; 0.03]), medium and high-risk level increasing 1.75 and 1.57 times respectively (Est.=0.028 and 0.15; 97.5% CI=[0.18 and 0.01; 0.01 and 0.028]), cancer increasing 1.68 times (Est.=0.52, 97.5% CI=[0.44; 0.60]), depression increasing 1.25 times (Est.=0.25, 97.5% CI = 0.17; 0.32) and obstructive chronic pulmonary disease increasing 1.52 times the use of hospital care services (Est.= -0.42, 97.5% CI=[0.34; 0.51]). Factors associated negatively with the use of hospital care services were being institutionalized which decreased 0.59 times the utilisation (Est.= -0.52; 97.5% CI=[-0.72; -0.32]) and hypertension which decreased 0.79 times the utilisation (Est.= -0.23, 97.5% CI=[-0.31; -0.15]) (Table 6).

**Table 6:** Factors associated with the utilisation of services by people with diabetes

**Utilisation of primary care services**				
Variables	Est.	Confidence interval 2.5% 97.5%	Exp (Est.)	P
Palliative care	0.23	0.15 0.31	1.26	0.00
Primary caregiver	0.33	0.29 0.37	1.39	0.00
Medium risk level High risk level	0.23 0.06	0.19 0.26 0.00 0.13	1.58 0.20	0.00 0.05
Complexity weight *	0.02	0.02 0.02	1.02	0.00
Chronic heart failure	0.20	0.15 0.24	1.22	0.00
Polymedicated	0.25	0.22 0.29	1.28	0.00
x^2^ (7) = 3615.02, *p* = 0.00 Pseudo-R2 (Cragg-Uhler) = 0.97. Pseudo-R2 (McFadden) = 0.19. AIC = 15278.62. BIC = 15317.92 * Quantitative variable				
**Utilisation of hospital care services**				
Variables	Est.	Confidence interval 2.5% 97.5%	Exp (Est.)	P
Institutionalised	-0.52	-0.72 − 0.32	0.59	0.00
Medium risk level High risk level index	0.28 0.15	0.18 0.37 0.01 0.28	1.75 1.57	0.00 0.03
Complexity index *	0.02	0.02 0.03	1.02	0.00
Cancer	0.52	0.44 0.60	1.68	0.00
Depression	0.25	0.17 0.32	1.28	0.00
Hypertension	-0.23	-0.31 -0.15	0.79	0.00
Obstructive chronic pulmonary disease	0.42	0.34 0.51	1.52	0.00
x^2^ (8) = 1065.94, *p* = 0.00 Pseudo-R2 (Cragg-Uhler) = 0.97. Pseudo-R2 (McFadden) = 0.19. AIC = 4624.40. BIC = 4664.41 * Quantitative variable				

## Discussion

Among the chronic patients belonging to the health centre, almost 11% had diabetes. They had a lower percentage of women, higher mean age and greater immobility and needs for care than patients without diabetes.

These people with diabetes had greater complexity and higher percentages of medium- and high-risk levels, as well as twice as many chronic diseases and greater multimorbidity. Their use of primary and hospital care services was very high and was mainly associated with functional factors related to the need for care and clinical factors regarding AMG risk level and complexity, some comorbidities and polymedication.

### Sociodemographic, functional, and clinical characteristics of people with diabetes

The percentage of people with diabetes in our study resembles those provided in other Spanish populations, such as the Prisma study, which indicated a prevalence of 11.1%, and the Di@bet study, with a prevalence of 13.8% [8, 9]. Also was within the range of other international studies which found a global prevalence of approximately 9.3%: 4.5% in Africa, 9.2% in Europe, 16.2% in the Middle East and North Africa, 11.9% in North America and the Caribbean, 14% in South and Central America, 8.7% in Southeast Asia and 11.9% in the Western Pacific [1].

The average age of the chronic nondiabetic patients was 54 years, compared to the average age of the people with diabetes, which stood at 70 years, with the highest-risk patients being older than the lower-risk patients. Estimation of age among other studies regarding adults with diabetes showed an average age between 75 and 79 years [8, 9, 31] and is expected to increase by 2030 [1]. Regarding the differences between sexes, there was predominance in the female sex in people without diabetes compared to people with diabetes. This study shows a slight predominance in the female sex with diabetes, which opposed other studies showing that diabetes was more frequent among males [9, 31, 32]. However, there was a predominance of female with diabetes older than 75 years compared to male patients. This could be explained by the fact that the global female population is older, according to the latest data in Spain [33].

In relation to their risk level, half of the patients were identified as low risk in the group without diabetes respect to people with diabetes, which meant a much higher percentage of medium- and high-risk patients with diabetes. Regarding multimorbidity, it was significantly higher in people with diabetes (94,4%) than in people without diabetes (5,2%). These data are higher than those of other cohorts of people with diabetes, which showed that 90% had at least one additional chronic condition [34].

In the data collected, the average number of chronic diseases presented in people with diabetes was 4.7, highlighting cardiovascular comorbidities such as hypertension, dyslipidaemia, dysrhythmias, coronary heart disease or heart failure. Other frequently associated noncardiovascular diseases were obesity, thyroid disorder, anxiety, depression, arthrosis, and anaemia. This is similar to other studies in which the most prominent comorbidities associated with diabetes are cardiovascular diseases, such as arrhythmias, arterial hypertension, coronary angioplasty, chronic heart failure or noncardiovascular diseases, such as chronic kidney failure, anaemia, cancer, liver disease, chronic obstructive pulmonary disease, cataracts or hypothyroidism [5, 34–36]. It is remarkable how the prevalence and number of these diseases increased according to the level of risk, as has been seen in other studies of patients relating chronic diseases and risk levels [6, 37]. The use of polypharmacy was much higher in people with diabetes than in people without diabetes. Approximately 90% of the high-risk people with diabetes in the study were polymedicated, compared to 15% in the group of low-risk patients. This coincides with other studies in which polymedication was associated with higher risk patients presenting greater comorbidities, hospitalisations and problems related to medication [38–41]. Therefore, establishing a specific plan in polymedicated people with diabetes is essential to prevent complications, reduce the utilisation of health services and ensure correct compliance with the therapeutic regimen.

Data from our study support how people with diabetes have a higher percentage of immobility, institutionalization and needs for care due to their functional impairment, greater multimorbidity and polymedication compared to people without diabetes. This is also reflected in the literature where it has been observed that these people with diabetes are associated with more care needs due to comorbidities and complications that chronically establish and aggravate their clinical prognosis and quality of life [42–45]. Identifying these characteristics of people with diabetes is critical for health care professionals to provide holistic and evidence-based care, focused on the patient situation and needs [46, 47].

### Use of health care services

In the study, a high number of contacts with the health system was observed, as has been seen in other studies of people with diabetes [18, 48, 49]. The percentage of patients who used primary care health services was higher than those who used hospital care, which is in line with other studies that estimate that chronic diseases generate 80% of primary care consultations and 60% of hospital admissions [22]. Consequently, the average number of annual contacts was higher in primary care health services than in hospital care services [17, 50].

All types of contacts with primary care were higher in females and older than 75 years old people with diabetes and increased according to the level of risk. These findings were similar to those from Coderch et al. [51], who found that the individuals classified as high risk corresponded to a higher total health consumption. The form of contact most used by people with diabetes at all levels of risk in primary care was the face-to-face event. Telephone consultation was twice as common in women and tripled as much in patients older than 75 years old. High-risk patients, as well as patients over the age of 75, required more telephone contacts and more home visits than those at medium and low risk. The professional most contacted was the family medicine doctor at all risk levels, both sexes, and age groups, followed by the nurses. It is remarkable how people with diabetes over 75 years of age had much greater contact with doctors and nurses than those under 75 years of age. This increase in contacts could be explained by the fact that they are patients with more complexity and comorbidities. This is observed in studies such as that of Martín-Fernández et al. [52], who observed that each new chronic condition was associated with an average increase in the number of family medicine doctors and nursing consultations. In terms of hospital care, the most used services were outpatient visits followed by emergency department visits and day-care visits, with hospitalisations being the less common contact with hospital care. High-risk patients had more contacts than medium- and low-risk patients. Men and people ≤ 75 years old had greater utilisation of hospital care services than women and those aged > 75 years, in contrast to findings related to the use of primary care services [53]. This difference is probably due to sociocultural facts in which women have more awareness of prevention and disease control at the primary care level than men, who, probably in the context of more cardiovascular risk factors and lower life expectancy, wait until they experience serious situations that require hospital care [54, 55]. The factors that best explain the greater utilisation of health-care services by elderly women versus men could be the number of chronic diseases and health-related quality of life [56].

This study is based on data from 2015 to 2016, so service utilisation may have changed in Madrid since then, especially with the system-wide changes with COVID-19 pandemic. Telephone consultations and home visits has been increased after that period because adequate material and human resources have been improved to facilitate this type of care. These forms of contact have demonstrated to be effective and necessary in order to improve patient care, especially those who have more risk and complexity. However, face to face primary and hospital care contacts have been reduced during pandemic times, although they have remained high and experiencing huge backlogs as observed in more recent studies [57–59].

In the multivariate analysis, it was observed how the use of services in both primary and hospital care was much influenced by AMG complexity index and medium and high-risk level, as observed in other studies with patients with multimorbidity or chronic complex patients (often equated with medium and high-risk patients) whose use of health care services could be multiplied by 6, compared to less complex patients with only a chronic disease [60–62]. These high-risk patients with diabetes are a challenge faced by the health system, since they usually constitute a complex and vulnerable population, with functional impairment, polypharmacy, frailty and a worse quality of life, associated with a high consumption of health resources [16–18, 31, 34, 45]. In primary care, the need for primary caregivers was related to higher utilisation of health services, and this could be explained by the fact that they are responsible for a very important part of patient care and accompany them in their use of health services. This is also reflected in studies that show that much of the total time dedicated annually in Spain to health care is devoted by caregivers [63]. This imposes a great physical, mental and social burden on the caregiver because it not only involves chronic patient care but also a processing of information, learning about the disease and overcoming the possible obstacles encountered in the process of care, which can mean an overuse of health services due the need for more support from health professionals [17, 18, 49]. The need for palliative care was also related to the utilisation of health services, which is understandable, as in Spain, palliative care is mainly administered at the patient´s home by primary care health professionals [64]. Other factors that increased primary care use were comorbidities such as chronic heart failure, which may be because follow-up is very well defined in primary care in Spain, or polymedication, as the family medicine physician in Madrid is the one who included all the treatments of the patients in the electronic prescription. Other studies also show that these comorbidities and polymedication are factors related to health care utilisation in people with diabetes in primary care [5, 18, 41, 65, 66]. However, in hospital care, comorbidities such as cancer, Chronic obstructive pulmonary disease (COPD) and depression were associated with higher use, which can be explained by the fact that this follow-up is more commonly carried out by oncologists, pneumologists and psychiatrists at the hospital care level. This situation is also seen in other studies in relation to comorbidities in people with diabetes and the use of hospital care services [19, 20, 66, 67]. Factors such as being institutionalized were associated with less use of hospital services, which is explained by the fact that the follow-up of people with diabetes is mainly made by health professionals in nursing homes and primary care. This was also true for hypertension, which is a comorbidity predominantly followed in primary care settings.

This high burden of care for primary caregivers and health care professionals required by complex patients with diabetes makes the role of case management professionals increasingly necessary [52], and for this reason, the care strategy of “case management” for the most complex patients is being promoted throughout Spain, mainly through the nursing staff [42, 68–70]. This type of strategy is of vital importance for individual planning and coordination of care, as it improves the quality and continuity of care, as well as optimises the services offered and reduces the costs derived from fragmented care [31, 66, 68]. People with diabetes needs require personalised care and treatment plans that are focused on their specific context. Morbidity groupers such as AMGs help health care professionals to identify these patients and develop individualized interventions [51, 71].

### Strengths and limitations

Due to the cross-sectional design of the study, the associations could not be interpreted in terms of cause-effect. In addition, information biases related to coding mistakes or diagnostic errors could limit the ability of the electronic medical record to reflect real morbidity. However, the use of these secondary clinical databases allows us to work with complete populations instead of partial samples or volunteers. Also, this is real-world data, which provided a large volume of information from the population in real clinical practice conditions, which minimises possible selection and memory biases. The possible existence of patients who did not use primary and hospital public care services due to having double insurance and not being represented in the total population of the centre could alter the real prevalence. Nevertheless, this is unlikely since the proportion of people with a health care card in the Community of Madrid has reached 95%, with very few patients being left out of the system [72].

In relation to AMGs, some doubts have been raised about their transparency, although this is a common situation with other commercial morbidity groupers [15]. In addition, these classification tools have a clinical care and management purpose that takes into consideration morbidity but does not consider other factors, such as social or economic status. Despite this, AMGs have shown good health service utilisation predictive capacity, and their developers have demonstrated that AMGs are a useful tool that allows the detection of chronic patients in health centres and facilitates the study of their clinical and organisational aspects, such as the risk level based on morbidity and complexity as well as the variability in their consumption of resources. Even so, the Spanish Ministry of Health aims to apply it in the National Health System for the management of chronic patients [21, 27, 73, 74].

## Conclusions

People with diabetes were older, with important needs of care, multimorbidity and polypharmacy that increased in parallel with the patient’s level of risk and complexity. The utilisation of primary and hospital care services was very high, being more frequent in primary care than hospital care. They are principally associated with functional factors related to the need of care and with clinical factors such as AMG risk levels and complexity, some serious comorbidities and polymedication.

This study provides novel data on comorbidities and risk levels by AMG in people with diabetes, as well as their utilisation of health services and needs of care. These data support health professionals in understanding risk levels in people with diabetes and could help to improve coordination between primary care teams, hospitals and caregivers, which would optimise diabetes clinical management and reduce costs derived from their consumption of resources.

### Electronic supplementary material

Below is the link to the electronic supplementary material.

Supplementary Material 1

## Data Availability

Datasets generated and analysed during the current study are not publicly available due they belong to the Madrid Health Service, but are available from the corresponding author on reasonable request.
