# Supplementary material for: Comorbidities and use of health services in people with diabetes mellitus according to risk levels by adjusted morbidity groups
Source: BMC Endocr Disord. 2024 Jul 16;24:115. doi: 10.1186/s12902-024-01634-0 (PMC11251131; doi:10.1186/s12902-024-01634-0)
Supplement: Supplementary file 1 — Supplementary Material 1 [file 12902_2024_1634_MOESM1_ESM.pdf]

TITLE: Comorbidities and use of health services in people with diabetes mellitus according to risk levels by adjusted morbidity groups.

AUTHORS:

Jaime Barrio-Cortes (1,2,3) *

María Pilar Mateos-Carchenilla (3,4)

María Martínez-Cuevas (5)

María Teresa Beca-Martínez (3)

Elvira Herrera-Sancho (6)

Carmen López-Rodríguez (6)

María Ángeles Jaime-Sisó (6)

Montserrat Ruiz-López (7) (8)

(1) Foundation for Biosanitary Research and Innovation in Primary Care. Madrid. Spain.

(2) Primary Care Investigation Unit. Gerencia Asistencial de Atención Primaria. Madrid. Spain.

(3) Faculty of Health. Universidad Camilo José Cela. Madrid. Spain.

(4) V Centenario Healthcare Centre, Gerencia Asistencial de Atención Primaria. San Sebastián de los Reyes, Madrid, Spain.

(5) Fuencarral Healthcare Centre, Madrid Health Service, Madrid, Spain.

(6) Ciudad Jardín Healthcare Centre. Gerencia Asistencial de Atención Primaria. Madrid. Spain.

(7) Nursing School. Fundación Jiménez Diaz Hospital – Universidad Autónoma de Madrid. Madrid, Spain.

***Corresponding Author:** Jaime Barrio Cortes. MD, PhD. Address: Fundación para la Investigación e Innovación Biosanitaria en Atención Primaria. Av. de la Reina Victoria, 21, 6ª Planta, 28003. Madrid, Spain. Email: jaime.barrio@salud.madrid.org. Phone: (+34) 660117699.

Additional file 1. Types of chronic diseases considered by the Adjusted Morbidity Group (AMG) in the Community of Madrid at the time of data extraction

Alcoholism
Anaemia
Aorta aneurysm
Anxiety
Arthritis
Arthrosis
Asthma
Attention-Deficit/Hyperactivity Disorder (ADHD)
Bladder cancer
Breast cancer
Cardiopulmonary disease
Central nervous system cancer
Cervical cancer
Cirrhosis
Colon cancer
Dementia
Depression
Diabetes Mellitus
Dyslipidaemia
Dysrhythmias
Ear, nose and throat cancer
Endometrial cancer
Epilepsy
Gastrointestinal ulcer

Glaucoma
Heart chronic failure
Hepatoblastoma
Hodgkin/Other lymphomas
Human immunodeficiency virus (HIV)
Hyperlipidemia
Hypertension
Ischemic heart disease
Leukemia
Liver cancer
Lung cancer
Mental retardation
Multiple sclerosis
Obesity
Obstructive chronic pulmonary disease (OCPD)
Osteoarthritis
Osteoporosis
Pancreatic cancer
Parkinson
Prostate cancer
Renal cancer
Renal chronic failure
Retinoblastoma
Schizophrenia
Skin cancer
Soft tissues cancer
Stomach cancer
Stroke
Substance abuse
Testicle cancer
Thyroid cancer

Thyroid disorder
Ulcerative colitis
Valvular heart disease
Vasculitis
